# Structural basis underlying CAC RNA recognition by the RRM domain of dimeric RNA-binding protein RBPMS

**DOI:** 10.1017/S0033583515000207

**Published:** 2015-09-08

**Authors:** Marianna Teplova, Thalia A. Farazi, Thomas Tuschl, Dinshaw J. Patel

**Affiliations:** 1 Structural Biology Program, Memorial Sloan-Kettering Cancer Center, New York, NY 10065, USA; 2 Laboratory of RNA Molecular Biology, Howard Hughes Medical Institute, The Rockefeller University, New York, NY 10065, USA

**Keywords:** The RNA-binding protein with multiple splicing, RNA recognition motif, photoactivatable-ribonucleoside-enhanced crosslinking and immunoprecipitation, RNA recognition element

## Abstract

RNA-binding protein with multiple splicing (designated RBPMS) is a higher vertebrate mRNA-binding protein containing a single RNA recognition motif (RRM). RBPMS has been shown to be involved in mRNA transport, localization and stability, with key roles in axon guidance, smooth muscle plasticity, as well as regulation of cancer cell proliferation and migration. We report on structure-function studies of the RRM domain of RBPMS bound to a CAC-containing single-stranded RNA. These results provide insights into potential topologies of complexes formed by the RBPMS RRM domain and the tandem CAC repeat binding sites as detected by photoactivatable-ribonucleoside-enhanced crosslinking and immunoprecipitation. These studies establish that the RRM domain of RBPMS forms a symmetrical dimer in the free state, with each monomer binding sequence-specifically to all three nucleotides of a CAC segment in the RNA bound state. Structure-guided mutations within the dimerization and RNA-binding interfaces of RBPMS RRM on RNA complex formation resulted in both disruption of dimerization and a decrease in RNA-binding affinity as observed by size exclusion chromatography and isothermal titration calorimetry. As anticipated from biochemical binding studies, over-expression of dimerization or RNA-binding mutants of Flag-HA-tagged RBPMS were no longer able to track with stress granules in HEK293 cells, thereby documenting the deleterious effects of such mutations *in vivo*.

## Introduction

RNA recognition motifs (RRMs) constitute the most abundant RNA-binding domains in higher vertebrates that play diverse roles in post-transcriptional gene expression processes ranging from mRNA and rRNA processing to RNA transport, localization and stability (reviewed in Clery *et al*. 2008; Gerstberger *et al*. 2014; Lunde *et al*. 2007; Serganov & Patel, 2008). RRMs are known to participate in both protein–protein and protein–RNA interactions, with the RNA targets in the latter complexes ranging from single-stranded RNAs to loop residues within RNA stem-loop folds. The RNA-binding protein with multiple splicing (RBPMS) has been shown to play important roles in axon guidance in retinal ganglion cells (Hornberg *et al*. 2013), in the control of smooth muscle plasticity (Sagnol *et al*. 2014), oocyte polarity (Heim *et al*. 2014) and regulation of cancer cell proliferation and migration (Fu *et al*. 2015).

A systematic study of transcriptome-wide mRNA targets of the RRM domain of RBPMS using photoactivatable-ribonucleoside-enhanced crosslinking and immunoprecipitation (PAR-CLIP) has identified RNA targets composed of tandem CAC trinucleotide motifs separated by variable spacer segments (Farazi *et al*. 2014). This has opened the opportunity for structural studies of complexes of the RRM domain of RBPMS with CAC-containing RNA targets to identify the molecular basis underlying protein-RNA recognition and also the potential role of RRM dimerization in contributing to the recognition of tandem CAC-containing RNA target sites.

We have solved the X-ray crystallographic structure of the RRM domain of RBPMS in its free form and in complex with CAC-containing U**CAC**U RNA. All RBPMS RefSeq isoforms share the same RRM sequence. The structure of the complex reveals the specific recognition of the CAC motif by the RBPMS RRM domain, as well as the dimeric arrangement of the protein that could enable the dimer to bind a pair of tandem CAC elements separated by a spacer of sufficient nucleotide (nt) length. We have also investigated the impact of structure-guided dimerization and RNA-binding mutants of RBPMS RRM on RNA-binding affinity and oligomerization, as well as localization of RBPMS to cytoplasmic stress granules under oxidative stress conditions.

## Results

### Crystal structure of RBPMS in the free state

**Fig. 1:**
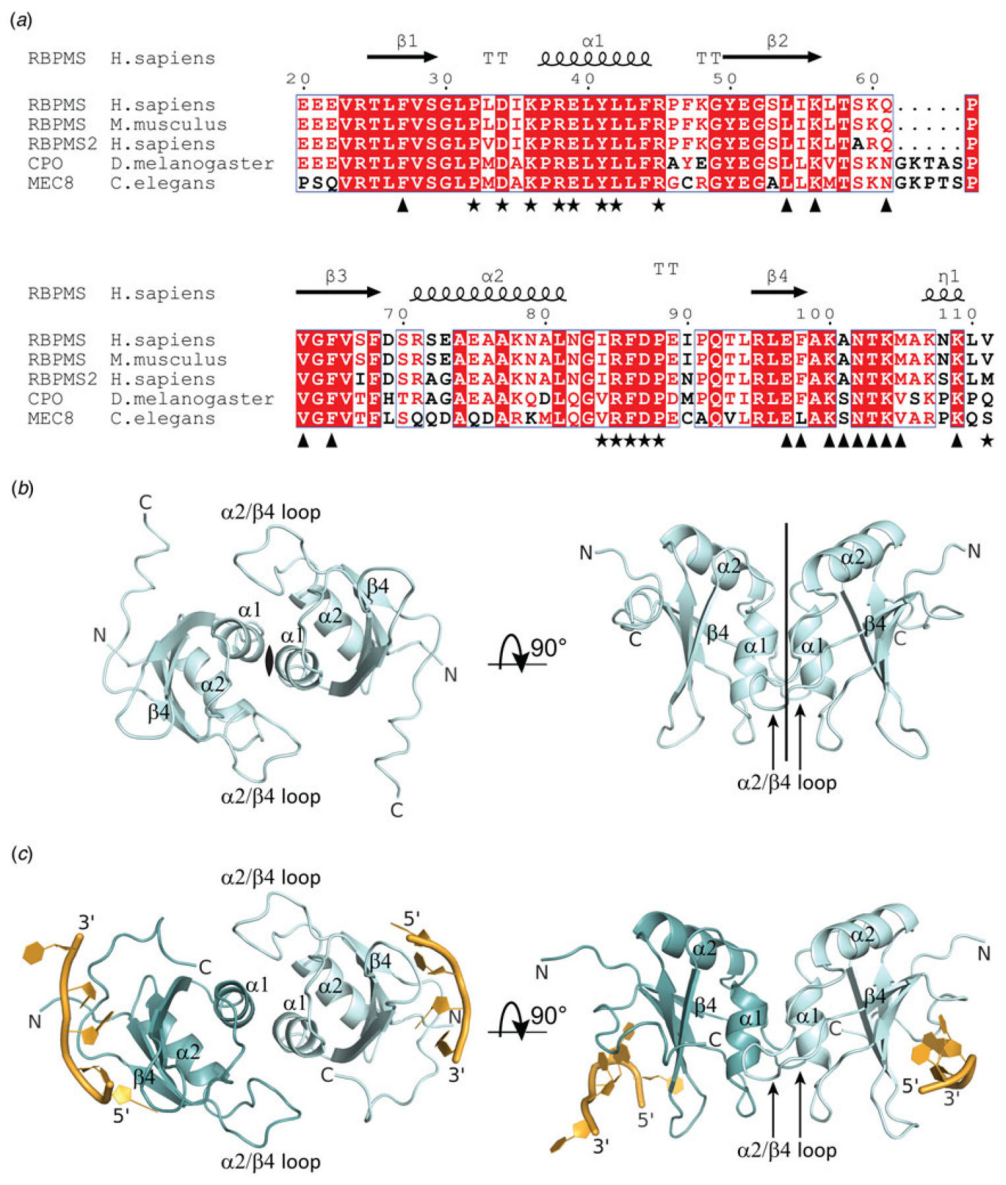
Crystal structures of RBPMS RRM homodimer in the free state and bound to RNA. (*a*) Structure-based sequence alignment of human RBPMS RRM with homologous sequences of RBPMS, RBPMS2, CPO and MEC8 from different metazoan species generated with ESPript (http://espript.ibcp.fr). Secondary structure elements of human RBPMS RRM are shown above the sequences. Homodimer interface residues are denoted by black asterisks, while residues involved in RNA binding are designated by triangles below the sequences. Residue numbering above the sequences corresponds to the human RBPMS. (*b*) Crystal structure of the RBPMS RRM homodimer. The two molecules related by crystallographic two-fold symmetry are shown in two orthogonal orientations. The secondary structure elements *α*-helix *α*1 and the loop between *α*-helix *α*2 and *β*-strand *β*4 involved in the dimerization are labeled. (*c*) Crystal structure of the RBPMS homodimer (cyan) bound to two UCACU RNA molecules (gold) in the crystallographic asymmetric unit shown in the same orientations as in panel (b). The secondary structure elements *α*1 and the loop between *α*2 and *β*4 involved in dimerization are labeled.

The RRM domain of RBPMS is conserved from humans to *D. melanogaster* (CPO protein) and *C. elegans* (MEC8 protein) (Fig. 1*a*), and based on this conservation, we expressed, purified and crystallized the RRM domain (residues 14–111) of human RBPMS in the free state and determined the structure at 1.79 Å resolution (x-ray statistics in Table 1). The structure was solved by single-wavelength anomalous dispersion (SAD) phasing on Se atoms using selenomethionine (SeMet)-labeled L81M mutant (Table 1). The crystals belong to the *C*222_1_ space group and contain two RRM molecules in the asymmetric unit (RRM molecules labeled mol A and mol B, Fig. S1a). The RRM of RBPMS adopts the classical RRM fold composed of a four-stranded antiparallel *β*-sheet packed against a pair of *α*-helices (Fig. 1*b*). The structure reveals two potential RRM dimeric arrangements, one within the asymmetric unit with minimal protein-protein intermolecular contacts mediated by two loop segments and the C-terminus (red dashed box in Fig. S1a), and another by crystallographic symmetry with extensive protein-protein intermolecular contacts mediated by residues of the first *α*-helix and adjacent loop region, as well as the loop segment between the second *α*-helix and the fourth *β*-strand (Fig. 1*b* and black dashed box in Fig. S1a). The latter interface (buried surface area = 1670 Å^2^) is associated with dimer formation according to the Complexation Significance Score 1·0 calculated with PDBePISA (Protein Interfaces, Surfaces and Assemblies, http://www.ebi.ac.uk/pdbe/prot_int/pistart.html).

### Crystal structure of RBPMS-RNA complex

Previous PAR-CLIP crosslinking studies of transcriptome-wide RNA binding sites in human embryonic kidney HEK293 cells had established that the RBPMS RRM domain specifically targets tandem CAC trinucleotide RNAs separated by a variable spacer region (Farazi *et al*. 2014). Providing a 5-nt RNA containing a central CAC trinucleotide segment, we obtained crystals and solved the 1.95 Å crystal structure of the RBPMS RRM domain bound to 5′-U^1^**C^2^A^3^C^4^**U^5^-3′ by molecular replacement using our solved structure of the RBPMS RRM L81M mutant in the free state as a search model (x-ray statistics in Table 1). The crystals belong to the *P*2_1_ space group and the asymmetric unit contains two RRM domains and two RNA molecules (Fig. 1*c*). The structure reveals the dimeric arrangement involving the *α*-helical surface of the RRM, by which two RRM molecules in the asymmetric unit are related by non-crystallographic symmetry (Fig. 1*c*), similar to the two crystal mates noted in the RNA-free RBPMS RRM domain structure (Fig. 1*b*). The *β*-sheet surface, in conjunction with the C-terminal loop on either side of the RRM homodimer, binds to the UCAC sequence of the RNA (Fig. 1*c*). Crystal contacts between the two UCACU RNA molecules (labeled Mol P and Mol Q_s_, Fig. S1b) bound to two separate RRMs (labeled Mol A and Mol B_s_, Fig. S1b) lead to the appearance of a pseudo-continuous 9 nt U**CAC**U**CAC**U RNA chain bound to two RRMs of two dimers in the crystal lattice.

Differences in the overall structures of the RNA-free and RNA-bound RBPMS are limited to the C-terminal loop (residues 103–111) that is involved in either protein-protein contacts in the RNA-free structure (Fig. S1a) or in protein–RNA interactions in the RNA-bound structure of the complex (Fig. S1b).

### Recognition by the RBPMS RRM domain of the CAC element of bound RNA

**Fig. 2:**
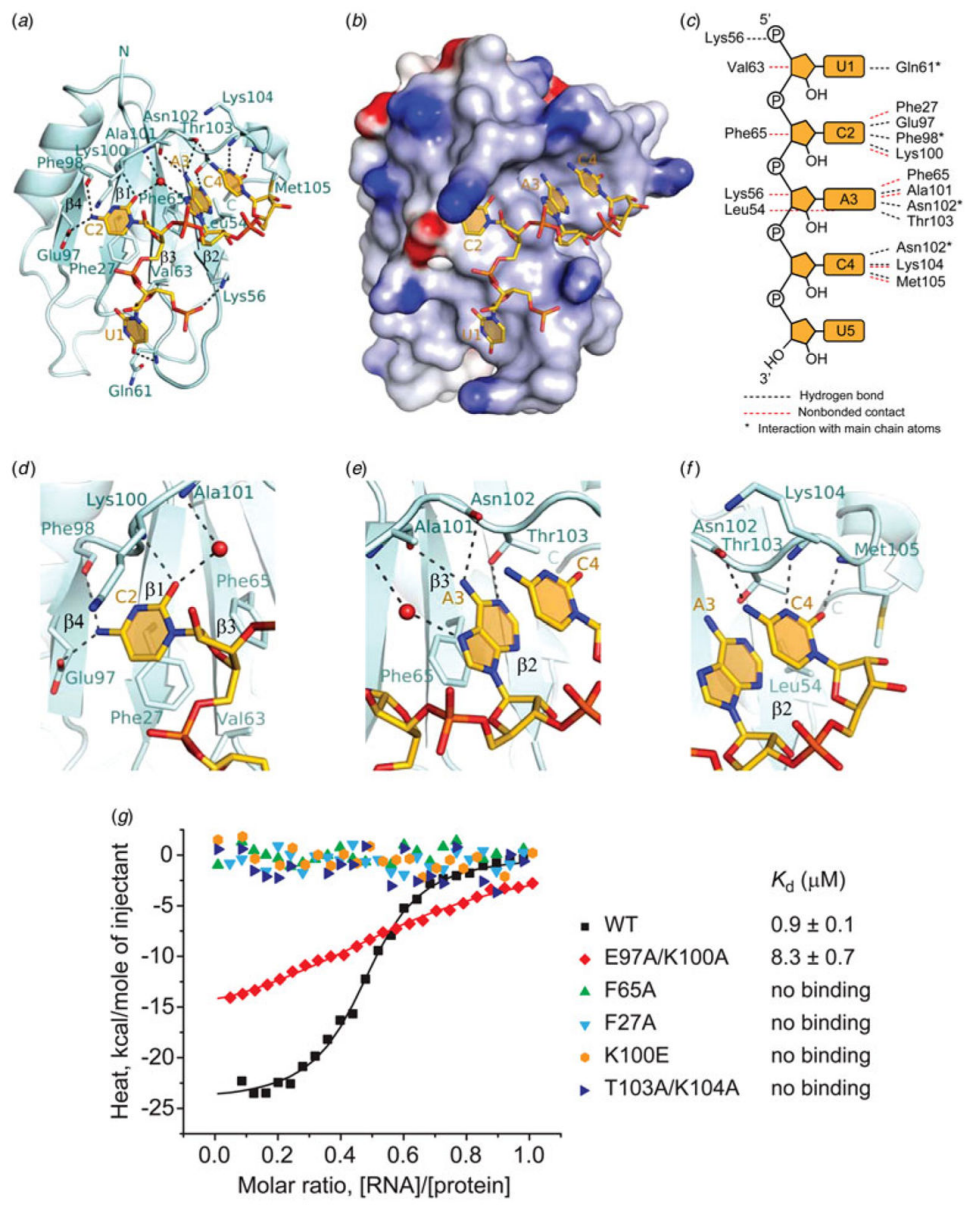
Protein-RNA intermolecular contacts in the RBPMS RRM-RNA complex. (*a*) Ribbon-and-stick representation of the complex containing one RRM molecule (cyan) of a homodimer bound to U1-C2-A3-C4 segment (gold) of the UCACU RNA oligonucleotide. The phosphorous, nitrogen and oxygen atoms are colored orange, blue and red, respectively. The four *β*-strands of the RRM *β*-sheet accommodating C2-A3-C4 motif are labeled. Intermolecular hydrogen bonds are shown as dashed lines. (*b*) An electrostatics surface view of RRM bound to U1-C2-A3-C4 (in stick representation) generated using the GRASP and PyMol programs. Basic and acidic regions of the protein appear in blue and red, with the intensity of the color being proportional to the local potential. (*c*) Schematic representation of protein–RNA interactions in the complex generated using the NUCPLOT software. Hydrogen-bonding and hydrophobic/stacking interactions between RNA bases and the sugar-phosphate backbone with RBPMS amino acid residues are shown by black and red dashed lines, respectively. Asterisks denote interactions involving protein main chain atoms. (*d*–*f*) Detailed view of the CAC motif recognition. (*d*) Hydrogen-bonding of the Watson–Crick edge of C2 with the backbone of RRM C-terminal loop and the side chain of Glu97 of *β*-strand *β*4. The base of C2 stacks with conserved Phe27 of *β*-strand *β*1 and Lys100 of the RRM C-terminal loop. (*e*) Hydrogen-bonding of the Watson–Crick edge of A3 with the backbone of Ala101-Asn102 and the side chain of Thr103 of the RRM C-terminal loop. The base of A3 stacks with conserved Phe65 of *β*-strand *β*3 in a parallel alignment with the C4 base. (*f*) Hydrogen-bonding of the Watson–Crick edge of C4 with the backbone of Asn102, Lys104 and Met105 of RRM C-terminal loop, and van der Waals interactions of C4 with the side chains of Lys104 and Met105. (*g*) ITC-binding curves of complex formation between the 17-nt G**CAC**UUUCAACUU**CAC**U ETF1 RNA target and the wild type RBPMS RRM (black squares), and the RBPMS RRM containing mutations of RNA contact residues E97A/K100A (red diamonds), F65A (green triangles), F27A (cyan reverse triangles), K100E (orange hexagons) and T103A/K104A (blue triangles). Solid lines represent nonlinear least-squares fit to the titration curve, with ΔH (binding enthalpy, kcal mol^−1^), *K*_a_ (association constant), and N (number of binding sites per monomer) as variable parameters. Calculated values for *K*_d_ (dissociation constant) are indicated.

The UCAC segment of the RNA is positioned over the four-stranded *β* sheet surface in the RBPMS RRM-RNA complex, with key intermolecular contributions by conserved aromatic amino acids (Phe27 and Phe65) projecting from the two central *β* strands of the RNA-binding surface of the RRM domain (Fig. 2*a*, *b*).

The U1 sugar is packed against Val63 of *β*3, while its base and phosphate interact with Gln61 and Lys56 of the loop between *β*2 and *β*3 strands (Fig. 2*a*, *c*). The C2, A3 and C4 bases stack over Phe27, Phe65 and Leu54, respectively, on *β* strands 1, 3 and 2, and are recognized sequence-specifically through extensive hydrogen bonding with both the side chain and backbone amino acid residues of *β*4 and the C-terminal loop (Fig. 2*a*, *c*–*f*). Recognition of C2 is mediated by hydrogen bonding interactions with the side chain of Glu97 and the backbone of Phe98 and Lys100, with Lys100 side chain forming a part of the binding pocket (Fig. 2*d*). Recognition of A3 is achieved through hydrogen bonding interactions of the base with the Ala101 and Asn102 backbone carbonyls and the Thr103 side chain (Fig. 2*e*). The C4 base is recognized via three hydrogen bonds with Asn102, Lys104 and Met105 backbone moieties, and with Thr103, Lys104, Met105 and Leu54 side chains that comprise the C4 binding pocket (Fig. 2*f*). The observed intramolecular stacking interactions involving A3 and C4 bases, as well as water-mediated contacts involving C2 and A3, additionally stabilize the bound RNA (Fig. 2*a*).

We note that the majority of the RBPMS RRM residues interacting with the RNA in the complex are strictly conserved in the corresponding RRMs of *D. melanogaster* CPO and *C. elegans* MEC8, with the exception of Ala101 and Met105 that are replaced by Ser and Val, respectively (Fig 1*a*).

### Impact of RBPMS RRM RNA-binding mutants on in vitro RNA-binding affinity

We used isothermal titration calorimetry (ITC) to measure RNA-binding affinities of RBPMS RRM mutations of key amino acids involved in CAC RNA recognition in the complex. We selected the PAR-CLIP-identified, natural target RNA segment G**CAC**UUUCAACUU**CAC**U RNA binding site located within the 3′ UTR of ETF1. This 17-nt RNA, which contains a pair of tandem CAC motifs spaced 9 nt apart, binds the wild-type RRM with a *K*_d_ of 0·9 *μ*M and approaching a 2 to 1 RRM to RNA binding stoichiometry (Fig. 2*g*). Mutation of conserved Phe27 and Phe65 residues, that are involved in stacking interactions with C2 and A3 bases in the complex, to Ala, resulted in undetectable binding (Fig. 2*g*). Further, Ala mutations of both Thr103 and Lys104 that form the C4 binding pocket (Fig. 2*f*), or reverse charge mutation of Lys100 to Glu, that participate in forming the C2 binding pocket (Fig. 2*d*), also resulted in undetectable binding (Fig. 2*g*). A double mutation of Glu97 and Lys100 to Ala, whose residues are also involved in C2 recognition (Fig. 2*d*), reduced binding affinity by an order of magnitude (Fig. 2*g*).

### RBPMS RRM homodimerization interface in the free protein and the RNA complex

**Fig. 3:**
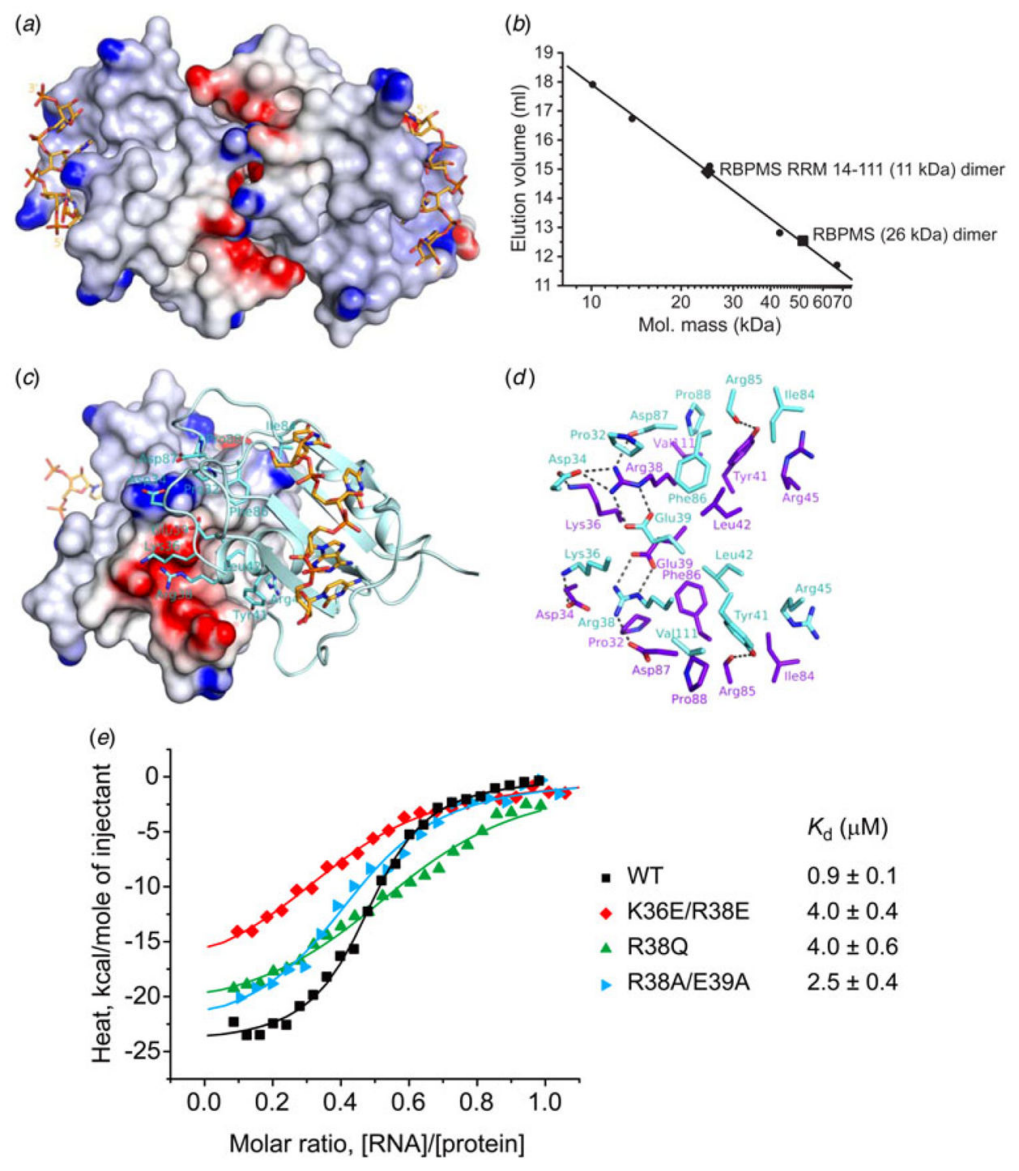
Homodimerization interface of the RBPMS RRM. (*a*) Electrostatic surface representation of the dimeric RBPMS RRM-RNA complex in the same view as shown in Fig. 1*c*, highlighting an electrostatic interaction between the basic and acidic residues along the dimer interface. (*b*) Gel-filtration elution volumes of the full-length RBPMS and RBPMS RRM (amino acids 11–114) plotted on the Superdex75 column calibration curve. (*c*) An electrostatic surface view of the dimer interface of molecule A with molecule B shown in a cyan ribbon and stick representation. Residues of molecule B involved in the dimer interface are labeled. Lys36 and Arg38 basic side chains interact with an acidic patch on the surface, and Glu39, Asp34 and Asp87 acidic side chains interact with a basic patch on the surface. (*d*) Details of the RBPMS homodimerization interface in the complex, highlighting interactions between residues involved in interfacial contacts. Residues of RRM molecules A and B are colored purple and cyan, respectively. The view is approximately the same in panel (c). (g) ITC-binding curves of complex formation between the 17-nt G**CAC**UUUCAACUU**CAC**U ETF1 RNA target and the wild type RBPMS RRM (black squares), and the RBPMS RRM containing mutations of dimerization interface residues K36E/R38E (red diamonds), R38Q (green triangles) and R38A/E39A (cyan triangles). Solid lines represent nonlinear least-squares fit to the titration curve, with ΔH (binding enthalpy, kcal mol^−1^), *K*_a_ (association constant), and N (number of binding sites per monomer) as variable parameters. Calculated values for *K*_d_ (dissociation constant) are indicated.

The RBPMS RRM dimeric arrangement within the asymmetric unit in the complex with RNA (Fig. 1*c*), as well as the arrangement of the two symmetry-related molecules in the RNA-free structure (Fig. 1*b*) reveal a common extensive homodimerization interface that is dominated by electrostatic and hydrophobic interactions (Fig. 3*a*).

We therefore studied homodimerization of the RBPMS RRM domain, as well as full-length RBPMS isoform A (ENSP00000318102) (Farazi *et al*. 2014), by size exclusion chromatography analysis in solution. The recombinant RBPMS RRM domain (11 kDa) and the full-length RBPMS protein (26 kDa) eluted with apparent molecular weights of 24·6 and 51·4 kDa, respectively, approximately the predicted molecular weights of homodimers (Figs 3*b* and S2a).

The homodimer interface is formed by symmetric interactions of the residues located on the *α*-helix 1 and the loops between *β*1 and *α*1, and between *α*2 and *β*4 (marked with asterisks in Fig. 1*a*). We observe key interfacial electrostatic interactions between two positively-charged residues (Lys36 and Arg38) and three negatively-charged residues (Asp34, Glu39 and Asp87) resulting in the formation of 10 salt bridges that contribute to homodimer formation (Fig. 3*c*, *d*). In addition, interfacial hydrophobic contacts involving Leu42, Tyr41 and Arg45 lying on *α*1-helix and Phe86 and Ile84 of the *α*2/*β*4 loop contribute to a hydrophobic core at the dimer interface, additionally stabilized by an interfacial hydrogen bond between Tyr41 hydroxyl and Arg85 backbone (Fig. 3*c*, d).

### Impact of RBPMS RRM interfacial mutants on dimerization and RNA binding

We generated RBPMS RRM interfacial mutants designed to disrupt the dimerization interface of the RRM domain. To this end, we mutated key charged amino acids (Arg38, Glu39 and Lys36) lining the electrostatic surface of the dimer interface (Fig. 3*c*, *d*). We constructed Ala, neutral and reverse charge mutations, R38A/E39A, R38Q and K36E/R38E, and assessed the impact of these mutations on dimerization and RNA binding affinity. Size exclusion chromatography of all three mutants revealed elution volume shifts toward monomer formation (Fig. S2b, c). ITC binding curves showed a 3- to 4-fold reduction of RNA binding affinity for K36E/R38E, R38Q and R38A/E39A mutants compared with the wild type protein (Fig. 3*e*).

### Impact of RBPMS mutants on subcellular localization

**Fig. 4:**
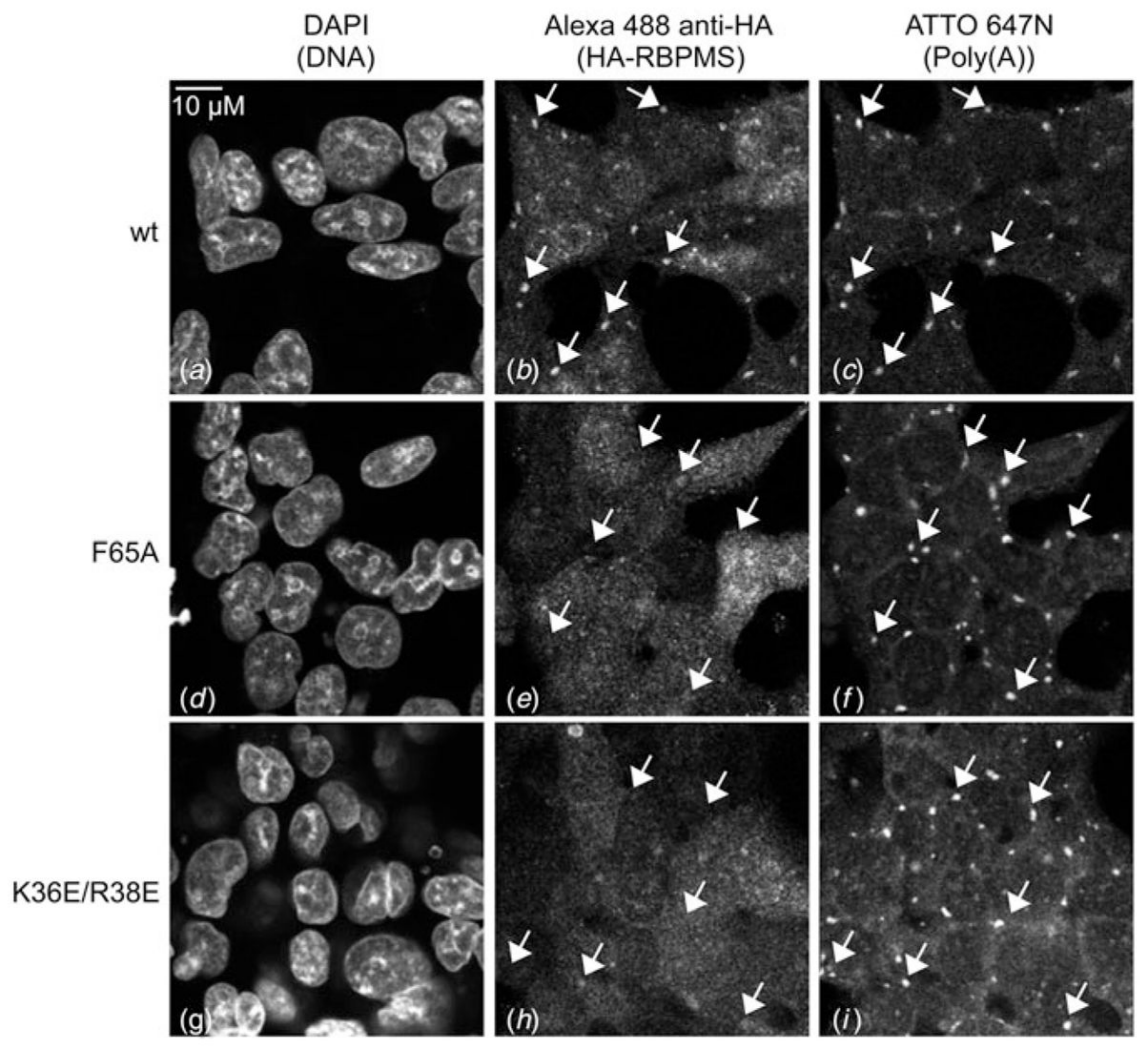
F65A RBPMS (d–f) and K36E/R38E RBPMS (g–i) display decreased localization to cytoplasmic stress granules after 400 *μ*M arsenite administration, compared with wild type RBPMS (a–c). Arrows point to representative stress granules in each image. Similar results were obtained for R38Q RBPMS and K100E RBPMS (Fig. S3).

To investigate the impact of the RNA-binding and dimerization mutations on *in vivo* RBPMS mRNA binding, we generated stable HEK293 cell lines inducibly expressing Flag-HA-tagged R38Q, K36E/R38E, F65A, K100E, as well as wild-type full-length RBPMS isoform A (ENSP00000318102). We had previously shown that wild type Flag-HA-tagged RBPMS co-localized with poly(A) RNA in cytoplasmic granules after oxidative stress treatment using 400 *μ*M arsenite, similarly to the known stress granule marker and mRNA-binding protein G3BP1 (Farazi *et al*. 2014). Following RNA fluorescence hybridization for detecting polyA mRNA and immunohistochemistry detection of the HA-tagged protein in HEK293 cells, RBPMS RNA-binding mutant (F65A, K100E) and dimerization mutant (R38Q, K36E/R38E) showed reduced colocalization to stress granules compared with wild type RBPMS (Fig. 4 and Fig. S3), which is indicative of reduced RNA-binding in live cells supporting the biochemical studies.

## Discussion

### Comparison of x-ray structure of homodimeric RBPMS RRM domain in the free and RNA-bound states

We showed that RBPMS RRM structures are very similar in the RNA-free and RNA-bound states (backbone rmsd of 0·75 Å for the entire domain, i.e. residues 23–102) with differences observed for the C-terminal loop segment (residues 103–111), that is involved in either protein-protein contacts in the RNA-free structure or in protein-RNA interactions in the complex (Fig. S4a). The relative orientation of the two RRMs in the crystal structure of free protein closely resembles the one observed in the crystal structure of RBPMS bound to RNA, defined by extensive symmetric electrostatic and hydrophobic interactions between the two RRMs. The small domain movement observed between the two crystal structures of the protein in the free and bound states (rmsd of 1·7 Å for the dimer alignment) likely results from crystal packing constraints between the two symmetry related molecules of the free protein homodimer (Fig. S4a). Therefore, the structure of RBPMS RRM in the complex with RNA, in which the two component RRMs of a homo-dimer belong to one crystallographic asymmetric unit, provides more reliable information on the relative orientation and interfacial contacts of the RBPMS homodimer.

### Comparison of x-ray structure of homodimeric RBPMS RRM domain in the free state with its NMR-derived RBPMS2 counterpart

The RBPMS RRM domain shares 90% amino acid identity with its paralog RBPMS2 RRM (Fig. 1*a*). An NMR structure has been reported for the RBPMS2 RRM domain in the free state (PDB: 2M9 K; Sagnol *et al*. 2014). Both the NMR and our reported x-ray structures have identified the same overall topology at the monomer level (rmsd = 1·5 Å), and to a lesser extent, at the homodimer level (rmsd = 2·6 Å), most likely due to crystal packing involving the two RRMs of RBPMS in the structure of the free protein (Fig. S4b).

The RBPMS RRM dimer interface is stabilized by electrostatic and hydrophobic interactions as outlined in the Results section and is virtually identical to RBPMS2 RRM dimer interface in solution (Sagnol *et al*. 2014). Notably, highly conserved interfacial charged residues when mutated (K36E/R38E and R38A/E39A dual mutants and R38Q single mutant) disrupt dimer formation (Fig. S2b, c) and result in reduced binding affinity to a natural target RNA comprising two binding sites (Fig. 3*e*).

The RBPMS and RBPMS2 RRM homodimerization interface (Fig. S4b) are distinct from any RRM homo- or hetero-dimerization interfaces reported in the PDB to date.

### Sequence specific recognition of the CAC RNA recognition element by the RBPMS RRM domain

Previous studies of RRM-RNA complexes have highlighted how the *β*-sheet of the RRM acts as a platform for RNA recognition, with base-specific recognition limited to two adjacent nucleotides (reviewed in Clery *et al*. 2008). By contrast, in our structure of the RBPMS RRM-RNA complex, base specific recognition is expanded to recognize a tri-nucleotide CAC motif, with base specific intermolecular hydrogen bonds involving the Watson–Crick edges of C2 (Fig. 2*d*), A3 (Fig. 2*e*) and C4 (Fig. 2*f*). Indeed, the majority of the intermolecular contacts are between the base edges of the RNA and the RRM domain (shown schematically in Fig. 2*c*) accounting for the high sequence-specificity of molecular recognition in the RBPMS-RNA complex. The complex is stabilized by intermolecular stacking interactions as observed between C2 and Phe27 (Fig. 2*d*), A3 and Phe65 (Fig. 2*e*) and intramolecular stacking interactions between A3 and C4 (Fig. 2*f*). Thus, it is not surprising that mutation of Phe27 and Phe65 to Ala results in complete loss in binding affinity (Fig. 2*g*). Similarly, mutational disruption of the C2-binding pocket (adjacent dual T103A/K104A mutations) and the C4-binding pocket (K104E mutant) also result in complete loss in binding affinity (Fig. 2*g*).

### Common intermolecular recognition of CAC element in RNA complexes with the RRMs of RBPMS and U1A proteins

Both NMR (Howe *et al*. 1994) and x-ray (Oubridge *et al*. 1994) structures have been reported for the complex formed between the RRM domain of the spliceosomal U1A protein bound to a stem-loop RNA. Sequence alignments of the RRM domains of RBPMS and U1A proteins (Fig. S5a) show similar distribution of *β*-strand and *α*-helices reflective of a common fold, as well as some conservation of the amino acid residues involved in recognition of the common CAC element. Notably, recognition of the C10-A11-C12 segment within the loop of the 21-nt stem-loop RNA by the RRM domain of monomeric U1A protein in the crystal structure of the complex (Fig. S5b, c) (Oubridge *et al*. 1994) has striking similarities with recognition of the C2-A3-C4 segment of the UCACU RNA by the RRM domain of dimeric RBPMS protein in the crystal structure of the complex reported in this study (Fig. 2*a*, b).

### Subcellular localization of RBPMS under stress conditions

In HEK293 cells RBPMS was suggested to be involved in mRNA transport and localization, having minimal effect on mRNA stability and splicing (Farazi *et al*. 2014). Analogous to many nucleocytoplasmic or cytoplasmic localized mRNA-binding proteins, RBPMS tracks under stress conditions to cytoplasmic stress granules. Furthermore, in frog oocytes, RBPMS2 is localized in the Balbiani body (Bb), a cytoplasmic mRNA-containing granule critical for oocyte polarity, during early oocyte development (Heim *et al*. 2014). In a rat retinal ganglion cell line, RBPMS is localized in neuronal granules transported in neurites during retinal differentiation, suggesting a role in retinal ganglion development (Furukawa *et al*. 2015). Consistent with this observation, Hornberg *et al*. had shown that in frog and zebrafish embryos RBPMS affects retinal ganglion synapse density and axon arbor formation (Hornberg *et al*. 2013). We observed that mutations in RBPMS RNA-binding or dimerization domains eliminated the ability to localize to stress granules in HEK293 cells, however, without any negative effects on stress granule formation as monitored by polyA mRNA accumulation using RNA-FISH, and therefore conclude that RBPMS is not critical for stress granule formation.

### Models for binding tandem CAC RNA segments by the dimeric RRE domains of RBPMS

Previous PAR-CLIP crosslinking studies established that the RBPMS RRM domain targeted tandem CAC trinucleotides separated by linkers that spanned 1–10 nt (Farazi *et al*. 2014). Our structure of the RBPMS-RNA (UCACU) complex (Figs 1*c*, 2*a*, *b* and S1b) provides insights into potential models for complexes involving tandem CAC trinucleotides separated by both short and long linkers.

**Fig. 5:**
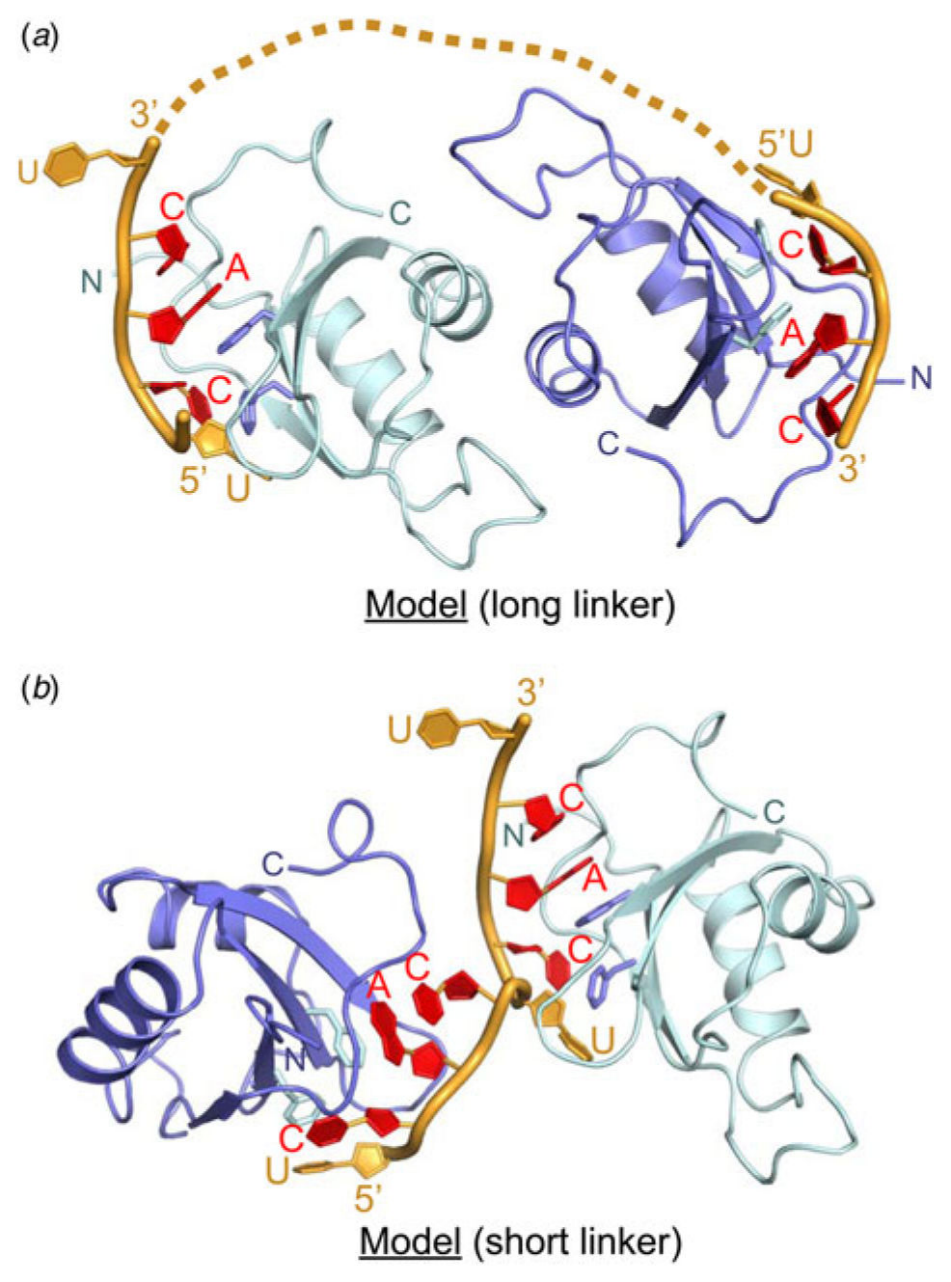
Models of interaction of RBPMS with RNA targets containing a pair of tandem CAC motifs separated by a linker based on the structure of the complex and crystal packing interactions shown in Fig. S1b. (*a*) Model of interaction of RBPMS homodimer with an RNA containing tandem CAC motifs spaced by a linker of sufficient length. (*b*) Model of two RBPMS monomers targeting two CAC motifs separated by a one-nucleotide linker. The dimerization surface of each RRM is available for dimer formation.

In the case of a long linker (between 8 and 10 nt), we propose a model where each CAC segment targets its binding site on the RBPMS RRM dimer exhibiting a large interface involving parallel alignments of the *α*1 helices (Fig. 5*a*), as seen in the crystal structure of the complex (Fig. 1*c*). The directionalities of the bound RNAs are such that a linker of approximately 8 nt or longer could readily connect the tandem pair of CAC elements. We were unable to structurally validate this proposed model since we failed to crystallize the complex containing two tandem CAC repeats separated by a 9 nt linker. Nevertheless, this model has common features with the corresponding model for binding of tandem CU repeat RNA to RRM3-RRM4 of PTB (Fig. S6a) (Oberstrass *et al*. 2005).

In the case of a short linker (between 1 and less than 8 nt), we propose a model where CAC segments target their binding sites on the separate RBPMS RRM domains (Fig. 5*b*), as seen in the crystal structure of the complex (crystal packing involving protein molecules *B* and *A*_s_ and RNA molecules *Q* and *P*_s_, Fig. S1b). We were unable to structurally validate this model since we also failed to crystallize the complex containing two tandem CAC repeats separated by 1–3 nt linkers. Nevertheless, this model has common features with the corresponding model for binding of UGUUUUUUU 9-nt RNA to RRM1-RRM2 of the Sex-lethal protein (Fig. S6b) (Handa *et al*. 1999), in which the 5′-UGU segment is bound by RRM2 and the UUU-3′ segment is bound by RRM1 in a similar manner to two UCA segments bound by two RBPMS RRMs (Fig. 5*b*). By contrast, the central UUU linker of the 9 nt sex-lethal protein RNA target interacts with RRM1 and has no structural analogs in RBPMS-RNA complex. It should be noted that although the relative orientations of the two RRMs are different in two models, they both allow binding a continuous RNA stretch (Figs 5*b* and S6b).

## Methods

### Protein expression and purification

The PCR-amplified cDNA fragments encoding the RRM domain of human RBPMS (14–111) were cloned into a modified pRSF-Duet1 (Novagen) vector encoding 6His-Sumo tag at N-terminus between the BamHI and XhoI restriction sites. The plasmid containing the DNA insert of interest was transformed into *Escherichia coli* strain BL21-CodonPlus (DE3)-RIL (Stratagene) grown in Luria-Bertrani (LB) medium supplemented with 50 mg ml^−1^ kanamycin. Single and double mutations of RBPMS L81M, F27A, F65A, K100E, R38Q, E97A/K100A, T103A/K104A, K36E/R38E and K36/E39A were introduced into the plasmid in one or two rounds of mutagenesis using the QuikChange II XL kit (Agilent) according to the manufacturer’s instructions. The SeMet substituted RBPMS L81M mutant was expressed by growing cells in a M9 minimal medium using a standard protocol to saturate the biosynthetic pathway for methionine production (Doublié, 1997). The recombinant protein expression was induced by 0·4 mM IPTG at 37 °C, followed by 12 h of incubation at 18 °C. The cell pellets were lysed using a French press and further clarified by centrifugation at 40 000 rpm. The proteins were then purified from the soluble fraction by a nickel-chelating affinity column HisTrap (GE Healthcare), followed by cleavage of the N-terminal His6-Sumo tag with the Ulp1 protease and additional purification by sequential chromatography on HisTrap, HiTrap Q HP and Superdex 75 columns (GE Healthcare). Protein purity was monitored on a polyacrylamide–SDS denatured gel.

### Crystallization, data collection and structure determination

RNA oligonucleotides were commercially synthesized (Dharmacon Research), deprotected and desalted according to the manufacturer’s instructions. Crystals of the RBPMS RRM and the complex of RBPMS RRM with 5′-UCACU-3′ RNA were grown by sitting-drop vapor diffusion. Crystallization conditions were determined with sparse matrix screens (Hampton Research, and Qiagen) using a Mosquito crystallization robot (TTP Labtech). The protein was crystallized by mixing equal volumes (0·2–0·4 ml) of 2 mM protein solution in 25 mM Tris-HCl pH 7·5, 0·5 M NaCl and reservoir solution containing 0·2 M lithium sulfate, 0·1 M Tris-HCl pH 8·5, 40% (v/v) PEG 400. The complex was crystallized by mixing equal volumes (0·2–0·4 ml) of 0·8 mM complex (1:1 protein to RNA molar ratio) solution in 25 mM Tris-HCl pH 7·5, 0·5 M NaCl and reservoir solution containing 0·1 M Bis-Tris-HCl pH 6·5, 28% (w/v) PEG 2000 MME. Droplets were equilibrated against 0·1 ml reservoirs at 20 °C. For data collection, crystals were flash-frozen (100 K) in liquid nitrogen. The data were collected on the 24-ID beamline at the Advanced Photon Source (APS) and processed by using HKL2000 (Otwinowski & Minor, 1997). The SAD data set was collected on SeMet RBPMS RRM L81M mutant crystal at a 0·97920 Å wavelength. Crystals of the free protein belonged to space group *C*222_1_, with two protein molecules per asymmetric unit. The crystals of the protein-RNA complex belonged to space group *P*2_1_ with two protein–RNA complexes per asymmetric unit. Crystal and diffraction data characteristics are summarized in Table 1.

The structure of the RBPMS RRM L81M mutant was determined by SAD phasing using the anomalous diffraction data collected at Se peak wavelength. A total of four Se sites were located using SHELEXD (Schneider & Sheldrick, 2002), and the AutoSol Wizard of the PHENIX package (Adams *et al*. 2010) was used for phasing and density modification. The initial experimental map showed clear density for most regions of the RRM. Iterative manual model building and refinement with phenix.refine produced the current model of the two RRMs (182 amino acids) in the asymmetric unit. The structure of the complex of RBPMS RRM bound to RNA was determined by molecular replacement with PHASER in the CCP4 suite using the coordinates of the free protein structure. The final structure refined with phenix. refine comprises two copies of the entire RRM domain (21–111) and two bound 5 nt RNA molecules. All protein residues in both structures are in allowed regions of the Ramachandran plot as evaluated in phenix.refine. Refinement statistics are given in Table 1.

### ITC measurements

ITC measurements were performed at 25 °C using an iTC200 (Microcal) calorimeter. Protein and RNA samples were dialyzed in 20 mM Tris-HCl pH 7·5 containing 100 mM NaCl. The protein concentration range in the cell of volume 200 ml was 0·03–0·05 mM. The RNA concentration range in the injection syringe of volume 60 ml was 0·26–0·5 mM. The data were analyzed with the Microcal ORIGIN software using a single site-binding model.

### Plasmid preparation for generation of stable cell lines

RBPMS mutations were introduced into the plasmid pFRT-TO-FLAG/HA-RBPMS_KDR. This plasmid was generated from pFRT-TO-FLAG/HA-RBPMS (available on http://www.addgene.org; 59388, Farazi *et al*. 2014) to introduce a silent mutation rendering the RBPMS transcript resistant to siRNA s21729 (Applied Biosystems). Mutations were introduced by one round of mutagenesis using a modified QuickChange kit protocol (Agilent), using KOD polymerase (EMD Millipore) and Top10 competent cells (Invitrogen). All plasmids are available on http://www.addgene.org.

### Cell culture

Flp-In HEK293 cells expressing Flag-HA-tagged wild-type and mutant RBPMS were prepared as previously described (Spitzer *et al*. 2013). Cells were maintained in DMEM containing 10% FBS, 2 mM glutamine, 15 *μ*g ml^−1^ blasticidin, and 100 *μ*g ml^−1^ hygromycin.

### Stress granule assay

HEK293 cells inducibly expressing Flag-HA-tagged wild type and mutant RBPMS were grown on Lab-Tek II chamber slides and induced with 1 *μ*g ml^−1^ doxycycline for 24 h. Arsenite was added to the cells at a final concentration of 400 *μ*M and incubated for 30 min at 37 °C. Slides were fixed and processed as previously described (Farazi *et al*. 2014). Images were recorded on an inverted TCS SP8 laser scanning confocal microscope (Leica) at the Rockefeller University Bio-Imaging Resource Center, using a 40X HC PL APO CS2 40X/1·10 Water objective, with the pinhole set to 1·00 Airy Unit. For Fig. 4 we used the following channels: (1) Excitation (Ex) 405 nm, Emission (Em) 451–486 nm, (2) Ex 492 nm, Em 500–550 nm, (3) Ex 640 nm, Em 648–710 nm. For Fig. S3 we used the following channels: (1) Ex 405 nm, Em 415–486 nm, (2) Ex 492 nm, Em 500–545 nm, (3) Ex 645 nm, Em 655–710 nm.

### Coordinates deposition

The atomic coordinates and structure factors for the RBPMS RRM L81M mutant and RBPMS RRM–RNA complex have been deposited in the Research Collaboratory for Structural Bioinformatics PDB with accession codes 5CYJ and 5DET, respectively.

## Supplementary Material

Supplemental

## Figures and Tables

**Table 1:** Crystallographic data and refinement statistics of the RBPMS RRM in the free state and bound to RNA

Protein/RNA	RBPMS (14–111) L81M	RBPMS (14–111)/UCACU
Data collection	SeMet	Native
Space group	*C*222_1_	*P*2_1_
Cell dimensions		
*a, b, c* (Å)	83·5, 90·9, 47·5	30·8, 90·23, 34·16
*a, b, g* (°)	90, 90, 90	90, 93·7, 90
Resolution (Å)	60–1·79	90–1·8
*R*_merge_	9·8 (99·1)	12·0 (77·5)
*I*/*σI*	17·0 (2·2)	10·3 (1·8)
Completeness (%)	99·9 (99·8)	99·4 (99·8)
Redundancy	8·5 (8·7)	6·3 (4·3)
Total unique	16298 (943)	17527(2559)
Refinement		
Resolution (Å)	60–1·79	20–1·95
No. reflections (free)	17 448 (882)	13 266 (656)
*R*_work_/*R*_free_	20·7/23·9	19·0/24·0
Residues		
Protein	182	186
RNA	–	9
H_2_O/cation	144/0	138/2
*B*-factors	28·7	28·0
R.m.s. deviations		
Bond lengths (Å)	0·009	0·008
Bond angles (°)	1·3	1·2
