# Supplementary material for: Structural basis underlying CAC RNA recognition by the RRM domain of dimeric RNA-binding protein RBPMS
Source: Q Rev Biophys. Author manuscript; Available in PMC 2016 Sep 27. (PMC4783296; doi:10.1017/S0033583515000207)
Supplement: Supplemental [file NIHMS738293-supplement-Supplemental.pdf]

Structural basis underlying CAC RNA recognition by the RRM domain of dimeric RNA-binding protein RBPMS

Marianna Teplova[1*], Thalia A. Farazi[2*], Thomas Tuschl[2] and Dinshaw J. Patel[1]

[1]Structural Biology Program
Memorial Sloan-Kettering Cancer Center
New York, NY, 10065, USA

[2]Laboratory of RNA Molecular Biology
Howard Hughes Medical Institute
The Rockefeller University
New York, NY, 10065, USA

* equal contribution authors

Supplementary Material

SUPPLEMENTARY FIGURE CAPTIONS

Figure S1. Overall structures of RBPMS RRM and RBPMS RRM-RNA complex. (a) Cartoon representation of two asymmetric units, each containing two protein molecules Mol A (cyan) and Mol B (teal). The two distinct protein interfaces are highlighted by the red and black dashed boxes. The two orthogonal views shown with the direction of crystallographic two-fold symmetry axis are the same as in Fig. 1b. (b) Cartoon representation of the crystallographic asymmetric unit depicted in Fig. 1c containing two protein molecules Mol A (cyan) and Mol B (teal) bound to two RNA molecules Mol P and Mol Q (gold), and the symmetry related contact RNA molecules Mol P_s and Mol Q_s bound to two protein molecules Mol A_s and Mol B_s. The terminal 3'U of Mol P overlaps with the 5'U of Mol Q_s in the crystal.

Figure S2. Elution profiles of RBPMS protein constructs on Superdex 75 10/30 column. (a) Full length RBPMS and RBPMS RRM (amino acids 14-111) profiles are labeled. (b) Wild type and mutant F27A, F65A, E97A/K100A, K100E, T103A/K104A, R38Q, K36E/R38E and K36/E39A RBPMS RRMs are colored black, blue, green, magenta, cyan, red, olive, dark blue, and yellow, respectively. (c) Peak positions and apparent molecular weights are tabulated for all tested constructs.

Figure S3. K36E/R38E (d-f), R38Q (g-i), F65A (j-i), and K100E (m-o) RBPMS display decreased localization to cytoplasmic stress granules after 400 μM arsenite administration, compared to wild type RBPMS (a-c). For assay conditions and description see Methods.

Figure S4. Comparison of the monomer structure and dimeric alignments of the crystal structure of RBPMS RRM in the RNA-free state with the corresponding RBPMS RRM structure in the complex with RNA, and with the solution structure of RBPMS2 RRM in the free state. (a) Overlay of the dimeric structures of RNA-free RRM (cyan) with RNA-bound RRM (orange) via one monomer molecule (left). While the two monomer structures overlay precisely except for the terminal loops, the relative orientation of the second monomer (right) of the homodimer differs in the free and bound structures. (b) Overlay of the dimeric crystal structure of RNA-free RBPMS RRM (cyan) with the solution structure of RNA-free RBPMS2 RRM (PDB ID 2M9K; purple) via one monomer molecule (left). While the two monomer

structures overlay well except for the loop regions, the relative orientation of the second monomer (right) of the homodimer differs in two structures.

Figure S5. Comparison of the crystal structure of RBPMS RRM bound to ssRNA with the crystal structure of U1A RRM1 bound to the stem-loop RNA (PDB ID 1URN. (a) Structure-based sequence alignment of human RBPMS RRM with human U1A RRM1 generated with ESPript (http://espript.ibcp.fr). Secondary structure elements of both RRMs are shown above the sequences. Residues involved in similar interactions with RNAs in both structures are denoted by triangles, with those that form hydrogen bonds with RNA bases by their backbones marked by reverse triangles. (b) Ribbon-and-stick representation of the structure of U1A RRM1 domain (grey) bound to an RNA loop (gold) containing C10-A11-C12 motif shown in the same orientation as RBPMS RRM bound to U1-C2-A3-C4 RNA element on Fig 2a. Positions of the bound CAC motifs in two structures overlay upon superposition of the two RRMs. U1A residues that contact A9-C10-A11-C12 RNA sequence are shown, with hydrogen bonds indicated by black dashed lines. (c) An electrostatics surface view of U1A RRM1 bound to an RNA hairpin loop shown in the same orientation as in panel (b). A9-C10-A11-C12 RNA segment is labeled. Basic and acidic regions of the protein appear in blue and red, with the intensity of the color being proportional to the local potential. The figure is generated using the GRASP and PyMol programs.

Figure S6. Examples of two modes of binding of tandem RRMs to RNAs, related to two modes of binding of RBPMS to RNA targets containing two CAC motifs shown on Fig. 5. (a) The structure of the PTB tandem RRM3 (cyan) and RRM4 (blue) connected by the linker (grey) bound to RNA (gold) (PDB ID 2ADC). The RRMs interact with one another to form a stable structural unit that presents the RNA-binding surfaces of the RRMs on opposite sides with bound RNA chains positioned in the opposite directions. The PTB RRM34-RNA structure is superimposed with the RBPMS RRM-RNA structure (Fig. 5a) via RRM3 and one of the component RRMs of the symmetrical RBPMS dimer (cyan). (b) The structure of the Sex-lethal tandem RRM1 (cyan) and RRM2 (blue) connected by the linker (grey) bound to RNA (gold) (PDB ID 1B7F). The two RRMs are positioned in a defined relative orientation to bind a continuous RNA stretch, with RRM1 binding to the 3’ end and RRM2 to the 5’ end of the RNA.

The Sex-lethal RRM12-RNA structure is superimposed with the RBPMS RRM-RNA structure (Fig. 5b) via RRM1 and the RBPMS RRM that interact with the 3’end of continues 9-nt RNA (cyan).

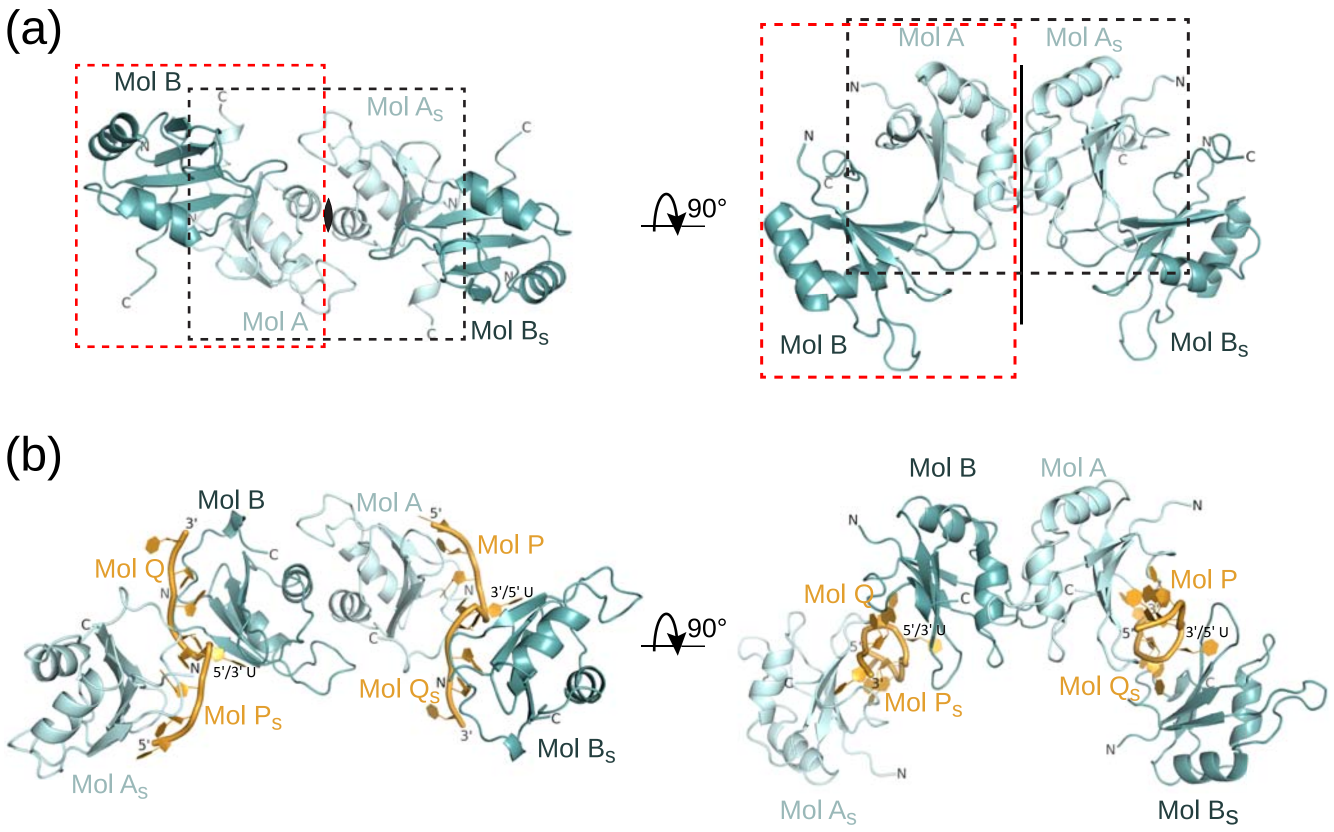

Figure S1

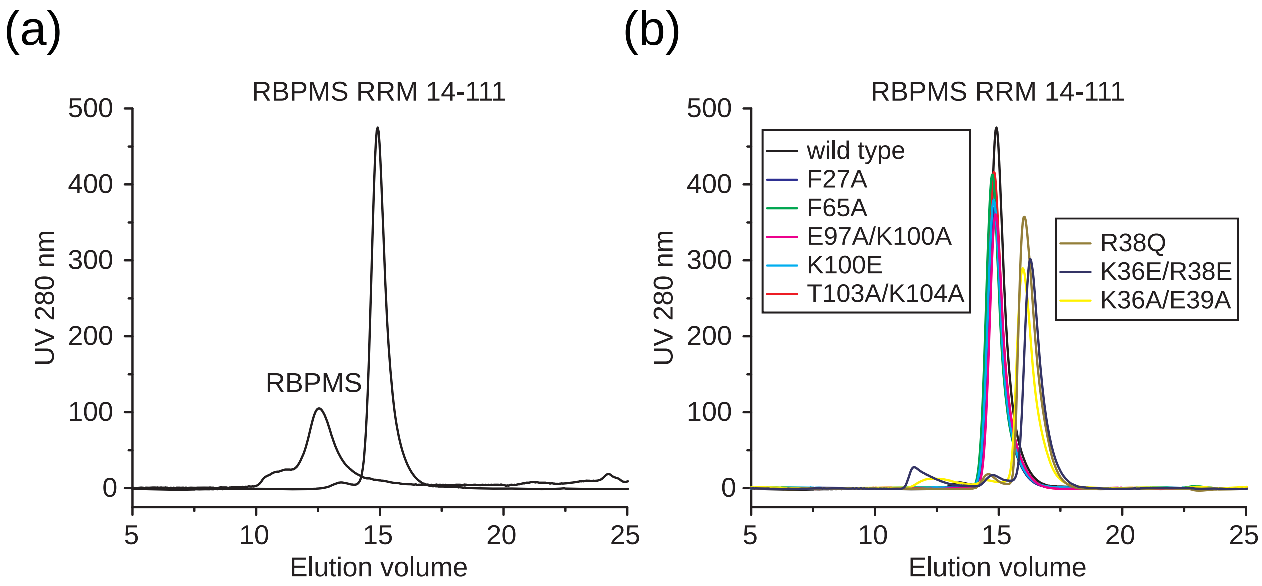

(c)

RBPMS	Elution volume, ml	Apparent MW, kDa
full length, MW 26kDa	12.54	51.4
14-111, MW 11 kDa		
wild type	14.9	24.6
RNA binding mutants		
F27A	14.83	25.04
F65A	14.75	25.4
E97A/K100A	14.87	24.95
K100E	14.8	25.4
T103A/K104A	14.82	25.2
Dimerization mutants		
R38Q	16.03	17.4
K36E/R38E	15.97	17.7
R38A/E39A	16.29	16.27

Figure S2

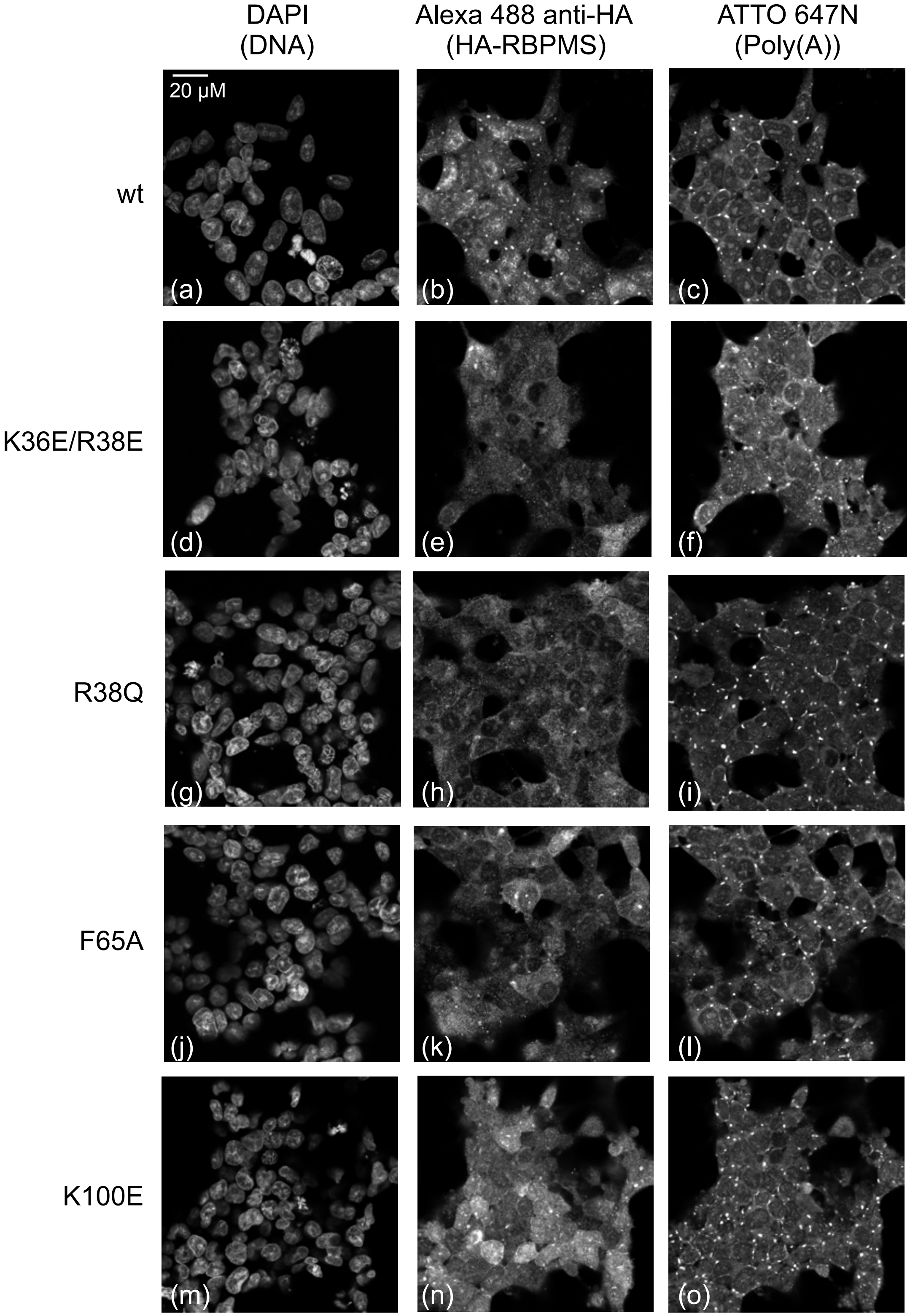
DAPI
(DNA)
Alexa 488 anti-HA
(HA-RBPMS)
ATTO 647N
(Poly(A))
20 μM
wt
K36E/R38E
R38Q
F65A
K100E
(a)
(b)
(c)
(d)
(e)
(f)
(g)
(h)
(i)
(j)
(k)
(l)
(m)
(n)
(o)

Figure S3

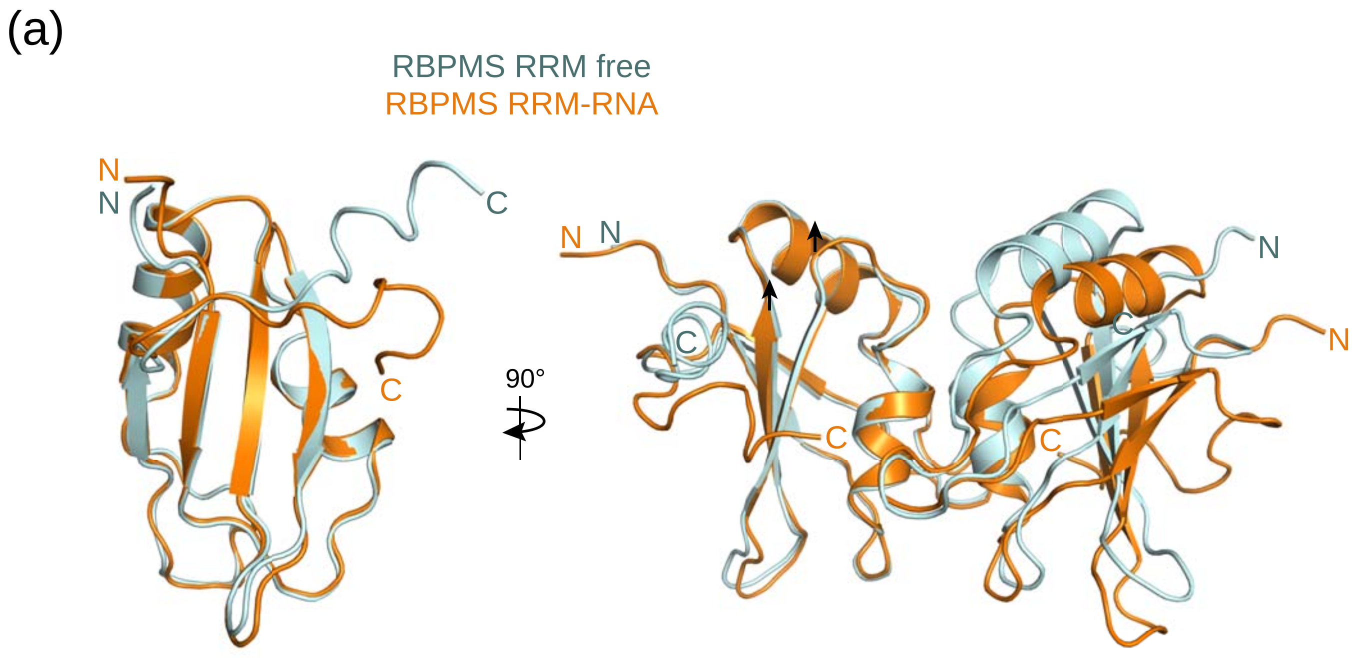

(a)
RBPMS RRM free
RBPMS RRM-RNA
N
N
C
C
90°
N
N
C
C
C
N
C
N

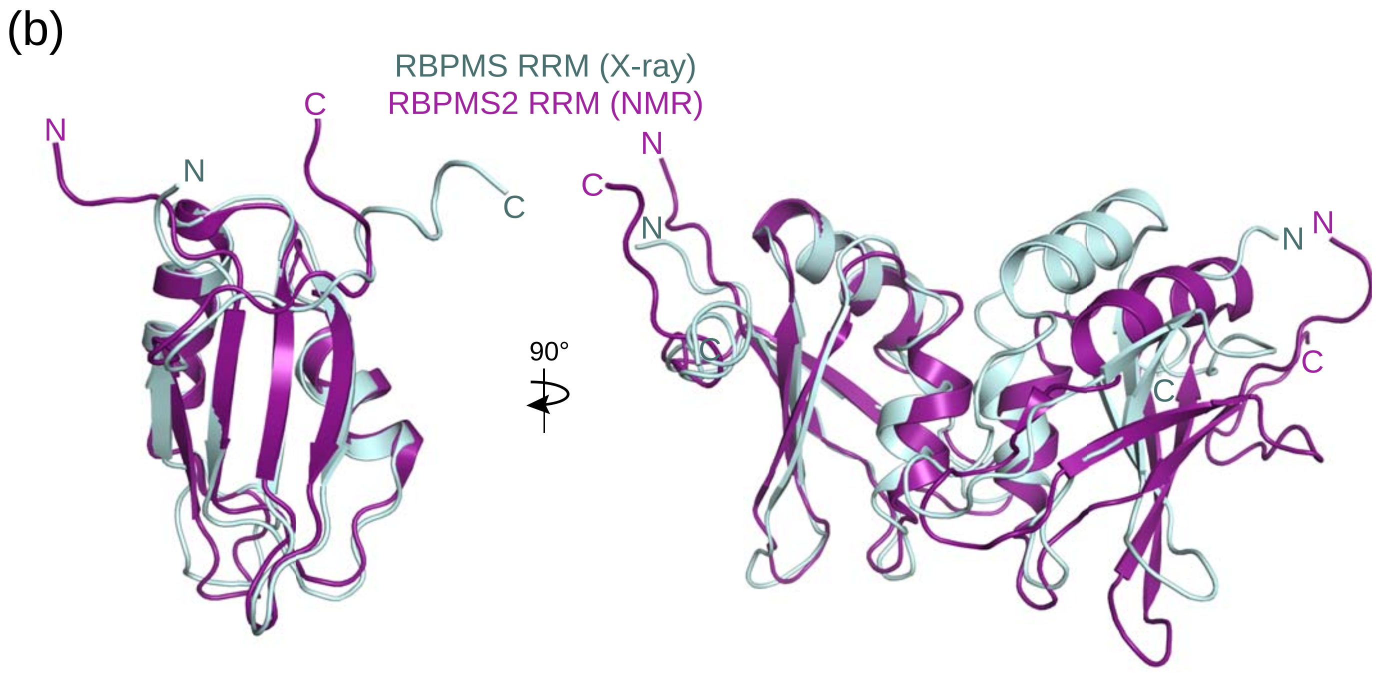

(b)
RBPMS RRM (X-ray)
RBPMS2 RRM (NMR)
N
C
N
C
90°
N
C
N
C
N
N
C
C

Figure S4

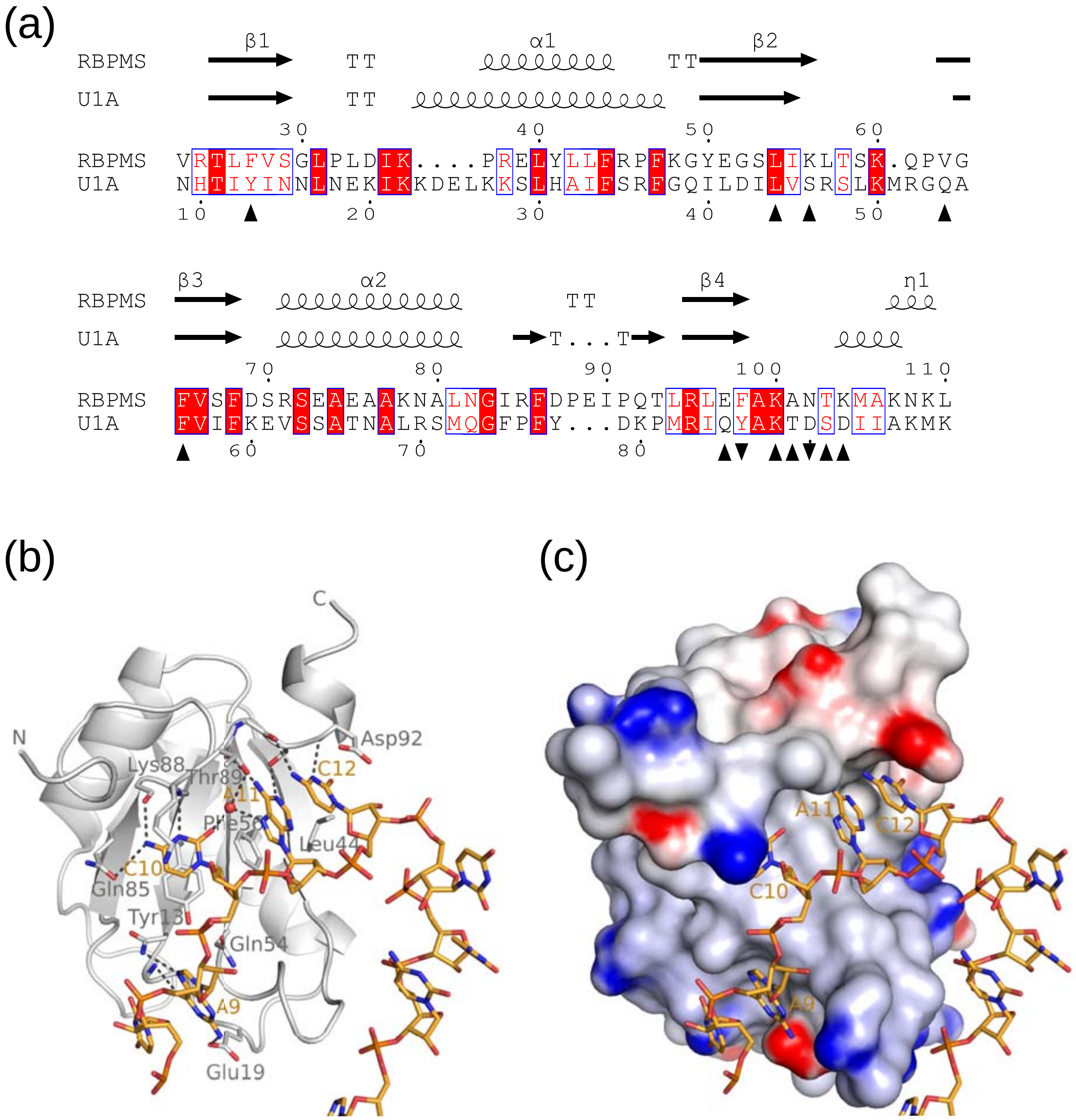
(a)
β1
α1
β2
RBPMS
TT
TT
U1A
TT
30
40
50
60
RBPMS VRTLFVSGLPLDIK....PRELYLLFRPFKGYEGSLIKLTSK.QPVG
U1A NHTIYINNLNEKIKKDELKKSLHAIFSRFGQILDILVSRSLKMRGQA
10
20
30
40
50
β3
α2
β4
η1
RBPMS
TT
U1A
T...T
70
80
90
100
110
RBPMS FVSFDSRSEAEAAKNALNGIRFDPEIPQTLRLEFAKANTKMAKNKL
U1A FVIFKEVSSATNALRSMQGFPFY...DKPMRIQYAKTDSDIIAKMK
60
70
80
(b)
C
N
Asp92
Lys88
Thr89
C12
A11
Phe56
Leu44
C10
Gln85
Tyr13
Gln54
A9
Glu19
(c)
A11
C12
C10
A9

Figure S5

(a)

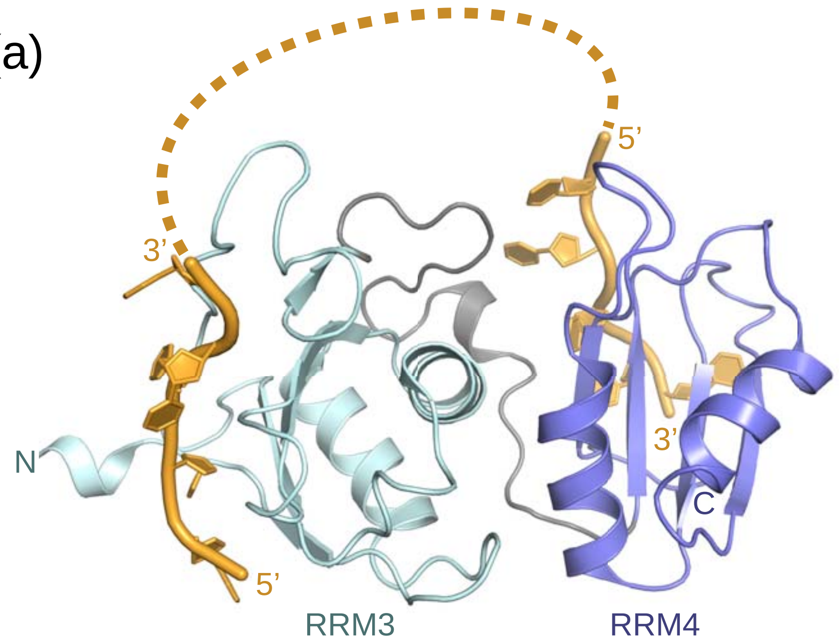

(b)

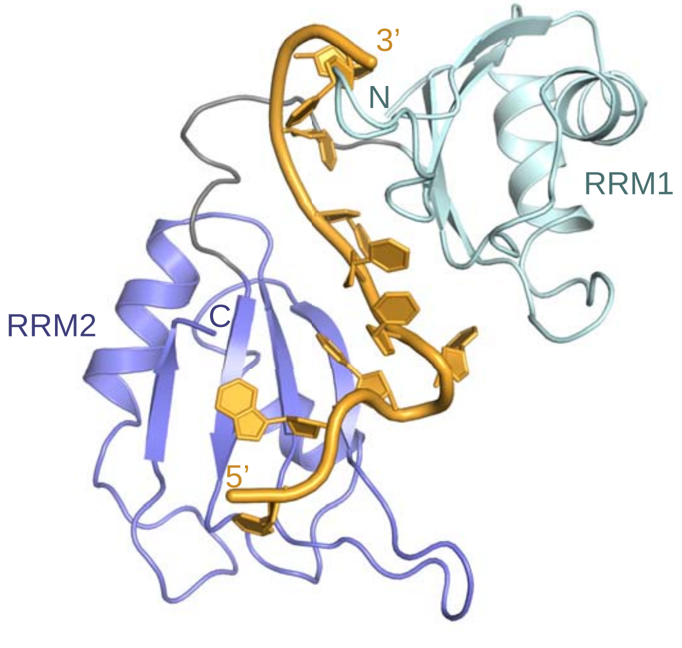

Figure S6
